# Identification of shared biomarkers in obesity and non-alcoholic fatty liver disease: a comprehensive analysis of Mendelian randomization and transcriptomic data

**DOI:** 10.3389/fnut.2026.1780686

**Published:** 2026-04-29

**Authors:** Jian Wang, Lei Zhang, Xinda Lu, Hui Niu, WenJun Zhao, Peng Ma, Jianli Han

**Affiliations:** Shanxi Bethune Hospital, Shanxi Academy of Medical Sciences, Third Hospital of Shanxi Medical University, Tongji Shanxi Hospital, Taiyuan, China

**Keywords:** biomarkers, Mendelian randomization, NAFLD, obesity, transcriptomic

## Abstract

**Introduction:**

Obesity and non-alcoholic fatty liver disease (NAFLD) are known to be closely interlinked; however, the mechanism of their interaction is unclear. This study sought to explore the causal relationship and shared biomarkers in obesity and NAFLD by combining Mendelian randomization (MR) and transcriptomic data.

**Methods:**

A bidirectional MR analysis was performed using summary statistics data from GWAS database to determine the causal relationship between obesity and NAFLD. Based on transcriptome data from GEO database, candidate genes were identified by combining differential expression analysis and expression level analysis. Subsequently, candidate genes were incorporated into least absolute shrinkage and selection operator (LASSO) and support vector machine-recursive feature elimination (SVM-RFE) algorithms to recognize shared biomarkers in obesity and NAFLD. The nomograms based on shared biomarkers were generated to predict the probability of developing obesity and NAFLD. Further, enrichment analysis and regulatory network construction were accomplished to excavate deeper into the molecular mechanism of obesity and NAFLD. Ultimately, expression level of shared biomarkers was validated through qRT-PCR.

**Rusults:**

MR analysis indicated significant causal effect of obesity on NAFLD (IVW: *P* = 0.034, OR = 1.252), while no significant causal effect of NAFLD on obesity (IVW: *P* > 0.05). Subsequently, based on the results of LASSO and SVM-RFE, we gained two shared biomarkers (NCAPH and IRS2) in obesity and NAFLD, and IRS2 was down-regulated and NCAPH was up-regulated in disease (obesity/NAFLD) samples. Receiver operating characteristic curves manifested that the efficacy of NCAPH and IRS2 in distinguishing between disease (obesity/NAFLD) and normal samples was all excellent. Besides, the nomograms constructed based on two shared biomarkers were reliable and had high reference value for predicting obesity and NAFLD. Enrichment analysis revealed that shared biomarkers could influence the development of both obesity and NAFLD via the “ECM-receptor interaction,” “Jak-STAT signaling pathway,” etc. In lncRNA-miRNA-mRNA network, lncRNAs (NEAT1, MALAT1, and FTX) could simultaneously regulate two shared biomarker via hsa-miR-493-5p. Ultimately, qRT-PCR results revealed that NCAPH was in line with the bioinformatics results.

**Conclusion:**

This study discovered causal effect of obesity on NAFLD and identified two shared biomarkers (NCAPH and IRS2) in two diseases, providing new insights for further explore the pathogenesis of both diseases.

## 1 Background

Obesity is a chronic metabolic disorder defined by excessive systemic fat accumulation. Per statistics from the World Health Organization (WHO), the global population of overweight and obese individuals has surpassed 2 billion, with 650 million classified as clinically obese (1). Its pathogenesis is driven by an imbalance between energy intake and expenditure, coupled with synergistic contributions from genetic susceptibility (e.g., FTO gene mutations), environmental factors (e.g., high-fat diet, sedentary behavior), and psychological components (2, 3). As a core component of metabolic syndrome, obesity directly precipitates type 2 diabetes mellitus (T2DM), non-alcoholic fatty liver disease (NAFLD), obstructive sleep apnea, and osteoarthritis. It also exhibits a significant association with various malignancies, including colorectal cancer and breast cancer (4). Collectively, obesity-related complications account for approximately 5 million premature deaths globally each year (5, 6).

NAFLD is a metabolism-associated hepatic disorder defined by excessive fat deposition in hepatocytes (hepatic fat content > 5%), after excluding alcohol consumption and other well-defined hepatic insults (7, 8). Its core etiology encompasses insulin resistance, dysregulated lipid metabolism, and oxidative stress, which interact synergistically with genetic susceptibility (e.g., PNPLA3 gene mutations) and environmental factors (e.g., high-calorie diet, sedentary lifestyle) (9). NAFLD frequently coexists with obesity and other cardiometabolic conditions, such as insulin resistance, T2DM, dyslipidemia, hypertension, and atherosclerosis. Owing to its intimate link with metabolism, it has been reclassified as metabolic dysfunction-associated fatty liver disease (MASLD). Obesity directly drives NAFLD progression via lipotoxicity (intracellular accumulation of free fatty acids), adipokine dysregulation (e.g., leptin resistance, reduced adiponectin), and chronic low-grade inflammation (elevated TNF-α and IL-6 levels). Visceral fat accumulation (waist circumference ≥ 90 cm in men and ≥ 80 cm in women) is more prone to inducing hepatic lipid metabolic dysfunction than generalized obesity; the prevalence of NAFLD reaches from 60%–95% in individuals with a body mass index (BMI) ≥ 30 kg/m^2^ (10). Despite a robust epidemiological association between obesity and NAFLD (odds ratio [OR] = 3–5), approximately 20% of non-obese individuals still develop NAFLD, while 30% of obese individuals show no hepatic steatosis, indicating a heterogeneous relationship between the two conditions (11). Currently, the specific molecular pathways (e.g., gut-liver axis, mitochondrial dysfunction, epigenetic regulation) that link obesity-related adipose tissue expansion to hepatocyte lipotoxicity remain incompletely elucidated, and the mechanisms underlying cross-organ metabolic crosstalk remain underinvestigated.

MR analysis serves as a robust approach to dissect causal relationships between exposures and outcomes. It typically leverages single nucleotide polymorphisms (SNPs) from genome-wide association study (GWAS) datasets as genetic instrumental variables to quantify the causal effects of exposures on outcomes (12). Compared with observational studies, MR analysis can mitigate the confounding biases inherent in traditional epidemiological designs. Previous MR studies have demonstrated that T2DM (OR = 1.58, 95% confidence interval [CI]: 1.42–1.77), dyslipidemia (a 1 mmol/L increase in triglycerides elevates NAFLD risk by 1.8-fold), and elevated serum alanine aminotransferase (ALT) levels exhibit causal associations with NAFLD (13, 14). Additionally, alcohol intake (β = 0.21, *P* = 3 × 10^–5^) and the abundance of specific gut microbiota taxa (e.g., Ruminococcus genus) may also participate in NAFLD pathogenesis. Despite the strong epidemiological correlation between obesity and NAFLD, existing MR studies provide insufficient genetic evidence to support a causal relationship. For instance, BMI-associated SNPs may indirectly affect hepatic fat deposition through non-adipose metabolic pathways (e.g., inflammatory pathways); cross-ethnic MR analyses consistently demonstrate substantial heterogeneity in effect estimates (I^2^ > 50%). Furthermore, the pleiotropy of obesity-related instrumental variables (e.g., the FTO gene, which concurrently regulates appetite and energy metabolism) has not been fully excluded. Thus, more refined two-sample MR analyses and multi-omics validation are urgently required to clarify the causal mechanisms underlying the obesity-NAFLD relationship.

Using obesity and NAFLD data from GWAS datasets, this study initially employed MR analysis to investigate the causal relationship between these two diseases. Subsequently, bioinformatics approaches were utilized to screen and identify shared biomarkers of obesity and NAFLD via transcriptomic data analysis. By exploring the functions and regulatory mechanisms of these biomarkers, this study further delineates the molecular links between obesity and NAFLD, with the aim of enhancing understanding of their common pathogenic mechanisms.

## 2 Materials and methods

### 2.1 Data collection

With respect to MR analysis, the Integrated Epidemiology Unit (IEU) Open genome-wide association study (GWAS) database provided all GWAS summary data, and individuals of European origin were the basis for this investigation. The obesity-related GWAS dataset (finn-b-E4_OBESITY) included 16,380,465 SNPs from 218,735 samples (8,908 obesity and 209,827 control samples), and the NAFLD-related GWAS dataset (finn-b-NAFLD) included 218,792 samples (894 NAFLD and 217,898 control samples) covering 16,380,466 SNPs. Both of the GWAS datasets are of the European population.

Additionally, transcriptomic data on gene expression profiles were collected from Gene Expression Omnibus (GEO) database^1^ utilizing the keywords “obesity” and “NAFLD.” Specifically, GSE59034 (16 obesity and 16 normal samples) and GSE135251 (206 NAFLD and 10 normal samples) were defined as the training sets, as well as GSE151839 (10 obesity and 10 normal samples) and GSE89632 (39 NAFLD and 24 normal samples) were defined as validation sets. Among them, the datasets GSE59034 and GSE135251 are from the European population (Germany), GSE89632 is from the European population (the United Kingdom), and GSE151839 is from the Asian population (China). Because the above data were obtained from public databases, no ethical review was required.

### 2.2 Data pre-processing for MR analysis

This study applied a bidirectional and two-sample MR design to investigate the bidirectional causal relationship between obesity and NAFLD. The obesity was the exposure factor and NAFLD was the outcome in the forward MR analysis, whereas the NAFLD was the exposure factor and obesity was the outcome in the reverse MR analysis. To ensure effective MR analysis, three basic assumptions should be satisfied: (i) instrumental variables (IVs) were strongly correlated with exposure factor; (ii) IVs should not be associated with confounders; and (iii) IVs could only influence outcome through exposure factors. Subsequently, we implemented a number of quality control steps to select eligible IVs based on three assumptions. Firstly, SNPs significantly associated with exposure factor were identified as IVs via “extract_instruments” function in “TwoSampleMR”-package (v 0.5.6) (15) with the threshold of *P* < 5*10^–6^. Next, SNPs with a linkage disequilibrium (LD) coefficient r^2^ > 0.001 and a physical distance of less than 10,000 kb were eliminated, to guarantee independence among IVs. Then, IVs significantly associated with the outcome were removed, and effect alleles and effect sizes were harmonized through the “harmonise_data” function of the “TwoSampleMR”-package. Finally, weak IVs (F-value < 10) were excluded from this study.

### 2.3 MR analysis and sensitivity analysis

In our study, five different methods were adopted to examine the causal relationship between obesity and NAFLD, including MR-Egger (16) weighted median (17), inverse variance weighted (IVW) (18), simple mode, and weighted mode (19), where IVW was utilized as the primary analytical method to establish the relationship (*P* < 0.05). Meanwhile, odds ratio (OR) value was calculated, and OR > 1 revealed that exposure factor was a risk factor, while OR < 1 revealed that exposure factor was a protective factor.

The sensitivity analysis was implemented through a variety of tests to evaluate the reliability of the MR analysis results. To begin with, Cochran’s Q test was executed to quantify the heterogeneity of IVs. If heterogeneity existed (*P* < 0.05), IVW-multiplicative random effects (IVW-MRE) approach was applied to correct the model. Immediately thereafter, the presence of horizontal pleiotropy was determined via MR-Egger intercept, with *P* > 0.05 implying the absence of horizontal pleiotropy. Eventually, leave-one-out (LOO) analysis was applied to identify the presence of abnormal SNP.

### 2.4 Differential expression analysis

Differential expression analysis was performed in the training sets (GSE59034 and GSE135251) via the “limma”-package (v 3.52.4) (20) and the “DESeq2”-package (v 1.36.0) (21), respectively, and the thresholds of the | log_2_FoldChange(FC)| > 0.5 and adj. *P* < 0.05 were employed to select the differentially expressed genes (DEGs) between obesity and normal samples in GSE59034 as well as the DEGs between NAFLD and normal samples in GSE135251. The volcano maps and heatmaps of DEGs were plotted via “ggplot2”-package (v 3.3.6) (22) and “ComplexHeatmap”-package (v 2.12.1) (23), respectively. Following this, the intersecting genes that were jointly up- and down-regulated by the DEGs in GSE59034 dataset and the DEGs in GSE135251 dataset were defined as shared DEGs in obesity and NAFLD, which were demonstrated through Venn diagrams via the online website.^2^

### 2.5 Functional annotation analysis and protein-protein interaction (PPI) analysis

Functional annotation analysis was carried out to reveal the biological functions of shared DEGs, especially in terms of biological processes and signaling pathways. Specifically, enrichment analysis for Gene Ontology (GO) and Kyoto Encyclopedia of Genes and Genomes (KEGG) was achieved via “clusterProfiler”-package (v 3.18.1) (24), and the cut-off value was *P* < 0.05. Further, with the aim of uncovering the role of target proteins at the systemic level, shared DEGs were introduced into the online Search Tool for the Retrieval of Interacting Genes (STRING)^3^ for constructing PPI network (interaction score > 0.4).

### 2.6 Machine learning algorithms

In the validation sets (GSE151839 and GSE89632), Wilcox-test was applied to compare differences in shared DEGs between disease (obesity/NAFLD) and normal groups. The genes that were significantly different (*P* < 0.05) between groups and consistent with the expression trend in the training sets (GSE59034 and GSE135251) were selected, and immediately these genes were recorded as candidate genes for subsequent analysis. Subsequently, in training sets (GSE59034 and GSE135251), a comprehensive analysis was accomplished to obtain shared biomarkers from candidate genes utilizing the least absolute shrinkage and selection operator (LASSO) and support vector machine-recursive feature elimination (SVM-RFE) algorithms. The “glmnet”-package (v 4.1-4) (25) and “e1071”-package (v 1.7-13) (26) were utilized to perform LASSO and SVM-RFE, respectively. Immediately thereafter, the intersecting genes were identified as shared biomarkers for the diagnosis of obesity and NAFLD by crossing the feature genes generated from the two algorithms.

### 2.7 Expression level analysis and diagnostic performance evaluation

To clarify the expression of shared biomarkers in obesity and NAFLD samples, the Wilcoxon-test was adopted to compare the expression levels of shared biomarkers between disease (obesity/NAFLD) and normal groups in the training sets (GSE59034 and GSE135251) and validation sets (GSE151839 and GSE89632). Afterward, the receiver operating characteristic (ROC) curves for each shared biomarker were plotted in the training sets (GSE59034 and GSE135251) and validation sets (GSE151839 and GSE89632) via the “pROC”-package (v 1.18.0) (27) to explore their ability to differentiate between disease (obesity/NAFLD) and normal samples.

### 2.8 Construction and evaluation of nomogram

Based on the above obtained shared biomarkers, nomograms were generated via “rms”-package (v 6.6-0) (28) in the training sets (GSE59034 and GSE135251) to predict the probability of developing obesity and NAFLD, respectively. Further, calibration curves were plotted to assess the reliability of the nomograms for diagnosing obesity and NAFLD, and decision curve analysis was completed to evaluate the clinical application value of the nomograms.

### 2.9 Enrichment analysis

To further explore the potential biological mechanisms involved in shared biomarkers in obesity and NAFLD, we completed gene set enrichment analysis (GSEA) in the training sets (GSE59034 and GSE135251), respectively. To begin with, the correlation coefficients between the shared biomarkers and the remaining genes were calculated and ranked, and followed by GSEA of the ranked genes via “clusterProfiler”-package (v 3.18.1) (24). The referenced gene was the KEGG gene set (c2.cp.kegg.v2023.1.Hs.symbols.gmt) from the Molecular Signatures Database (MsigDB),^4^ and the cut-off values for significance were set to *P* < 0.05 and | NES| > 1.

In this study, the co-expression and pathway networks of shared biomarkers were constructed using the online analysis application GeneMANIA,^5^ aiming to predict potential biological functions of shared biomarkers.

### 2.10 Chromosomal localization and subcellular localization analyses

To uncover the location of shared biomarkers on human chromosomes, the distribution of biomarkers on chromosomes was visualized using Circos website.^6^ It was well known that the biological functions of mRNAs were closely linked to their localization in cells. Therefore, in this study, subcellular localization analysis of shared biomarkers was carried out by the mRNALocater database.^7^

### 2.11 Construction of regulatory network

To investigate the molecular regulatory mechanisms of shared biomarkers in obesity and NAFLD, transcription factor (TF)-mRNA and lncRNA-miRNA-mRNA regulatory networks were constructed. Firstly, the ENCODE database^8^ was utilized to retrieve potential TFs regulating the shared biomarkers. Cytoscape software (v 3.9.1) (29) was employed to generate the TF-mRNA network. Afterward, the StarBase^9^ and miRDB^10^ databases were deployed to predict the target miRNAs of shared biomarkers, followed by the upstream lncRNAs of the miRNAs were obtained through StarBase and miRcode^11^ databases. Ultimately, visualization of lncRNA-miRNA-mRNA network utilizing “ggalluvial”-package (v 0.12.5).

### 2.12 Quantitative real time polymerase chain reaction (qRT-PCR)

The expression of shared biomarkers was validated by qRT-PCR. Total RNA was extracted from liver and greater omentum adipose tissue specimens of 5 obese patients with non-alcoholic fatty liver disease (NAFLD) and 5 standard control subjects using TRIzol reagent (Ambion, Austin, USA). Investigators collected samples from patients with obesity-related NAFLD and control individuals at the Shanxi Bethune Hospital. The diagnostic criteria for patients with obesity and non-alcoholic fatty liver disease (NAFLD) in this study were as follows. Obesity was diagnosed according to the Guidelines for the Prevention and Control of Overweight and Obesity in Chinese Adults, with a body mass index (BMI) cutoff value of ≥ 28.0 kg/m^2^. The BMI of enrolled patients ranged from 41.7 to 46.6 kg/m^2^, all of whom met the diagnostic criteria for obesity. NAFLD was diagnosed in accordance with the Guidelines for the Diagnosis and Treatment of Non-Alcoholic Fatty Liver Disease (2018 Revised Edition), combined with abdominal ultrasound findings showing moderate or moderate-to-severe fatty liver. All patients denied a history of alcohol consumption, and alcoholic liver disease and other etiologies that could cause fatty liver were excluded. Patients who fulfilled both the above diagnostic criteria for obesity and NAFLD were diagnosed as having obesity complicated with NAFLD. The clinical information is presented in Supplementary Table 1. This study received ethical approval from the Ethics Committee of Shanxi Bethune Hospital (SBQLL-2025-212, 2025-03-12). After extraction, the samples were allowed to stand for 15 min, and 1 μL of the samples was taken for concentration assay using Nanodrop N50. Then cDNA was synthesized by reverse transcription using the SweScript First Strand cDNA synthesis kit (Servicebio, Wuhan, China). Subsequently, the cDNA obtained from reverse transcription is diluted by a factor of 5 to 20 for qRT-PCR verification and the relative expression levels were calculated using the 2^–ΔΔCt^ method. Details of the primers, reaction systems, and amplification conditions are presented in Supplementary Tables 2–4.

### 2.13 Statistical analysis

In the present work, all statistical analyses involved were performed by R program (v 4.2.2). *P*-value < 0.05 was deemed statistically meaningful unless otherwise stated.

## 3 Results

### 3.1 The clinical baseline characteristics of the study participants

A total of 10 participants were included in the qRT-PCR validation cohort, consisting of 5 patients with obesity-related NAFLD and 5 healthy controls. The baseline characteristics of the two groups are summarized in Table 1. Significant differences were observed in BMI (*P* = 0.012) and ALT levels (*P* = 0.008), while no significant differences were found in age, AST, creatinine, urea, or gender distribution (all *P* > 0.05). Except for BMI and ALT—which are expected to differ due to disease status—other baseline variables showed no significant differences between groups, indicating acceptable comparability.

**TABLE 1:** The clinical baseline characteristics of the study participants.

Variables	Total (*n* = 10)	Experimental group (*n* = 5)	Control group (*n* = 5)	T/X^2^	*P*
Age, M (Q_1_, Q_3_)	35.50 (30.00, 43.25)	30.00 (30.00, 36.00)	45.00 (35.00, 59.00)	Z = −1.05	0.295
BMI, M (Q_1_, Q_3_)	32.60 (22.90, 42.77)	42.90 (42.40, 43.20)	22.70 (22.50, 23.50)	Z = −2.51	**0.012**
ALT, M (Q_1_, Q_3_)	33.55 (17.60, 59.45)	62.80 (49.40, 63.80)	15.10 (9.80, 25.10)	Z = −2.65	**0.008**
AST, M (Q_1_, Q_3_)	32.00 (25.85, 40.95)	43.70 (32.10, 57.50)	26.00 (21.00, 31.90)	Z = −1.67	0.095
Creatinine, M (Q_1_, Q_3_)	75.35 (71.25, 78.55)	74.10 (70.30, 79.10)	75.60 (75.10, 76.90)	Z = 0.00	1.000
Urea, M (Q_1_, Q_3_)	4.65 (4.10, 5.95)	4.60 (4.10, 6.20)	4.70 (4.10, 5.20)	Z = −0.21	0.833
Gender, *n* (%)				–	1.000
Male	3 (30.00)	2 (40.00)	1 (20.00)		
Female	7 (70.00)	3 (60.00)	4 (80.00)		

Z, Mann–Whitney test; –, Fisher exact; M, Median; Q_1_, 1st Quartile; Q_3_, 3st Quartile. Bold values indicate results with a statistically significant difference (*P* < 0.05) between the two groups.

### 3.2 The causal effect of obesity on NAFLD

**FIGURE 1:**
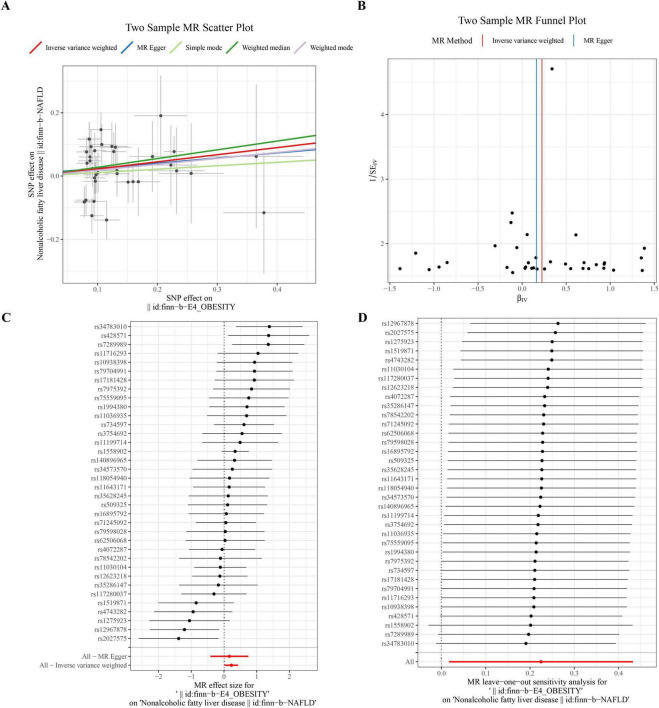
MR analysis of the causal link between obesity and NAFLD. **(A,B)** Scatter and Forest plots confirm obesity as a significant risk factor for NAFLD. **(C)** Funnel plot assessing potential bias. **(D)** Leave-one-out (LOO) sensitivity analysis confirming result robustness.

The results of the forward MR analysis were indicated in Table 2, where the significant causal effect of obesity on NAFLD (*P* = 0.034, OR = 1.252) was found in the IVW-MRE approach. In the meantime, it was proved that obesity was a risk factor for NAFLD based on the observation of OR values. Scatter plots, funnel plot, and forest plot were utilized to illustrate the data so that they could be understood more clearly. The notion that obesity was a risk factor for NAFLD was further reinforced by the positive slope of the lines in scatter plot and the MR effect size of surpassing 0 in forest plot, suggesting that obesity elevated the chance of getting NAFLD (Figures 1A,B). According to the funnel plot (Figure 1C), MR was in accordance with Mendel’s second law of random grouping.

**TABLE 2:** MR analysis of the causal effect of obesity on NAFLD.

Exposure	Outcome	Method	Nsnp	pval	OR
OBESITY	NAFLD	MR Egger	36	0.585	1.177
		Weighted median	36	0.053	1.318
		Inverse variance weighted (MRE)	36	0.034	1.252
		Simple mode	36	0.68	1.115
		Weighted mode	36	0.326	1.204

Furthermore, according to the Cochran’s Q test, significant heterogeneity (*P* > 0.05) was observed among the selected SNPs (Table 3); therefore, we chose the IVW-MRE model for correction. Horizontal pleiotropy tests indicated that the intercept of MR-Egger was less than 0.05 and the *P*-value was greater than 0.05 (Table 4), meaning that there was no horizontal pleiotropy. Ultimately, the LOO analysis revealed that removal of any individual SNP had no significant effect on the remaining SNP results, indicating that the MR analysis results were reliable and robust (Figure 1D).

**TABLE 3:** Heterogeneity test of the causal effect of obesity on NAFLD.

Exposure	Outcome	Method	Q	Q_df	Q_pval
OBESITY	NAFLD	MR Egger	51.519	34	0.028
		Inverse variance weighted	51.595	35	0.035

**TABLE 4:** Pleiotropy test of the causal effect of obesity on NAFLD.

Exposure	Outcome	egger_intercept	SE	pval	Presso_P
OBESITY	NAFLD	0.008	0.035	0.824	0.048

### 3.3 The causal effect of NAFLD on obesity

We also carried out a reverse MR analysis with NAFLD as the exposure factor and obesity as the outcome. As demonstrated in Table 5, there was no significant causal effect of NAFLD on obesity (IVW: *P* = 0.570). With regard to sensitivity analysis, it was proven that the results of the MR analysis were reliable. Scatter plot, forest plot, funnel plot, Cochran’s Q test, MR-Egger test, and LOO analysis results were presented in Supplementary file.

**TABLE 5:** MR analysis of the causal effect of NAFLD on obesity.

Exposure	Outcome	Method	Nsnp	pval	OR
NAFLD	OBESITY	MR Egger	12	0.469	0.973
		Weighted median	12	0.581	0.987
		Inverse variance weighted	12	0.57	1.009
		Simple mode	12	0.969	0.998
		Weighted mode	12	0.722	0.989

### 3.4 Totally 134 shared DEGs in obesity and NAFLD were acquired

**FIGURE 2:**
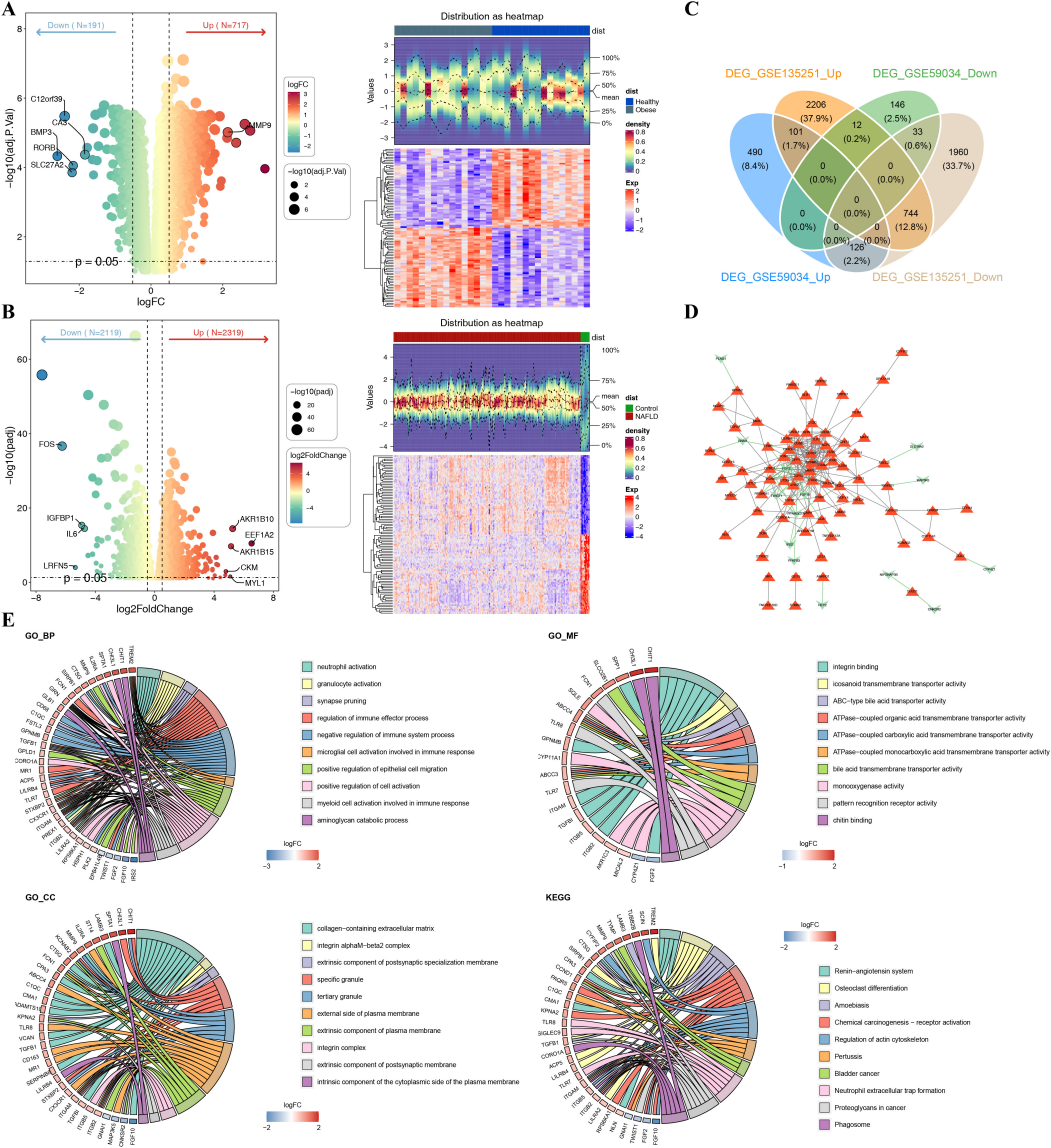
Identification of shared DEGs and functional enrichment. **(A,B)** Volcano plots and heatmaps of DEGs in obesity (GSE59034) and NAFLD (GSE135251). **(C)** Venn diagram identifying 134 shared genes (101 up-, 33 down-regulated). **(D)** PPI network of shared DEGs via STRING. **(E)** GO and KEGG analyses highlighting immune and metabolic pathway enrichment.

Differential expression analysis revealed that 908 DEGs (717 up-regulated and 191 down-regulated) between obesity and normal samples in GSE59034 as well as 4,438 DEGs (2,319 up-regulated and 2,119 down-regulated) between NAFLD and normal samples in GSE135251 were identified, respectively. Visualization of DEGs through volcano maps and heat maps (Figures 2A,B). Immediately following this, 134 shared DEGs (101 up-regulated and 33 down-regulated) were discovered using Venn diagrams by taking common up- and down-regulated genes of 908 DEGs in GSE59034 and 4,438 DEGs in GSE135251 (Figure 2C). The PPI network of shared DEGs was demonstrated in Figure 2D, and the network contained 92 nodes and 245 edges, of which MMP9, TGFB1, GPNMB and CD163 were the more important ones.

### 3.5 Shared DEGs were linked to cell activation and immune-related pathways

Further, 134 shared DEGs were incorporated into GO and KEGG enrichment analysis to investigate the biological mechanisms. The results demonstrated that these 134 genes were enriched into 1,091 entries, including “neutrophil activation,” “regulation of immune effector process,” “negative regulation of immune system process,” “microglial cell activation involved in immune response,” “neutrophil activation involved in immune response,” etc (Figure 2E). Meanwhile, under the KEGG analysis, 28 pathways were enriched (*P* < 0.05), such as “renin-angiotensin system,” “osteoclast differentiation,” “regulation of lipolysis in adipocytes,” “AMPK signaling pathway,” “FoxO signaling pathway,” etc (Figure 2E). These analyses highlighted the importance of cell activation and immune-related pathways in the pathogenesis of obesity and NAFLD.

### 3.6 NCAPH and IRS2 were shared biomarkers in obesity and NAFLD

**FIGURE 3:**
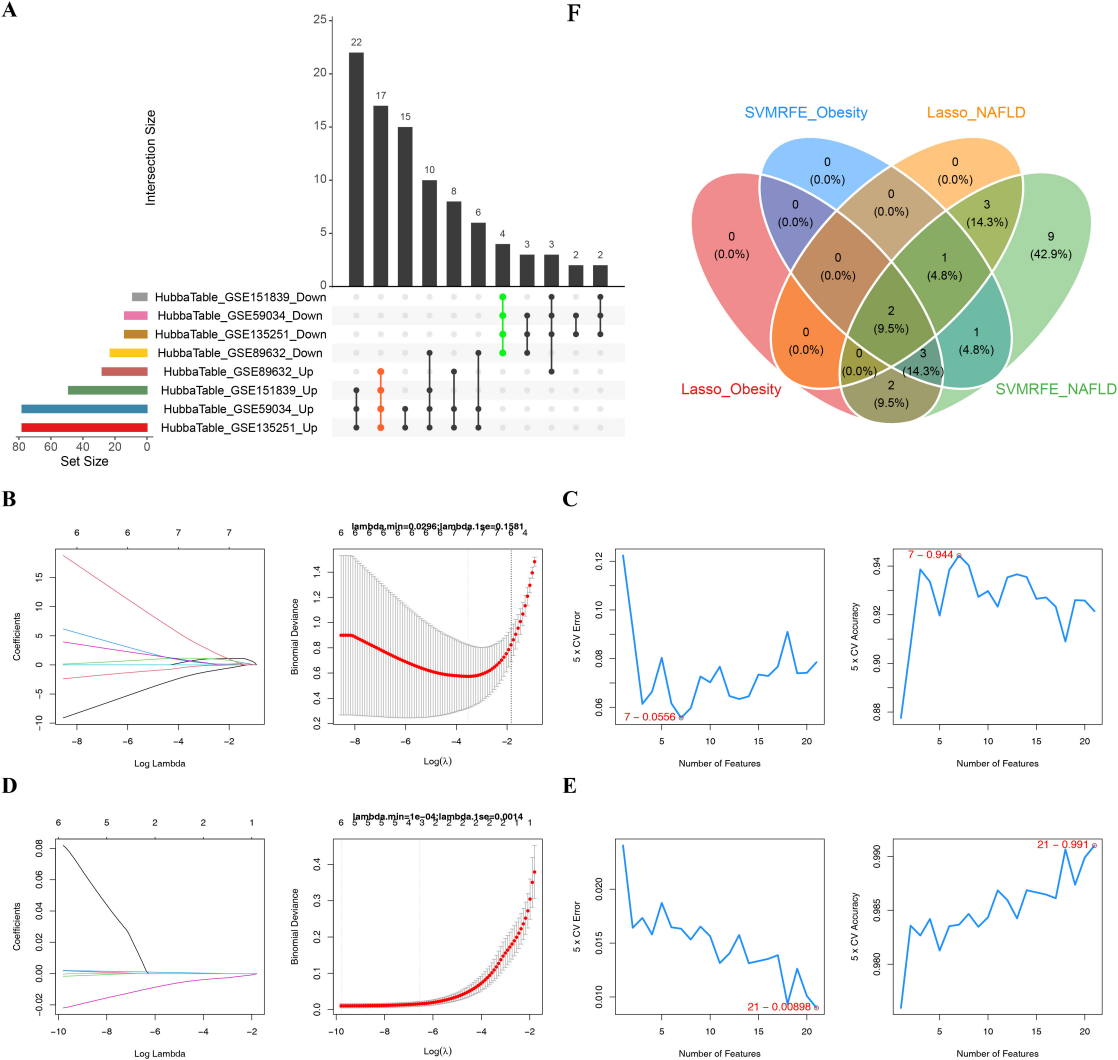
Biomarker selection via machine learning. **(A)** Intersection analysis of candidate DEGs. **(B)** In the OBESITY group, LASSO regression analysis was used to screen for characteristic genes. **(C)** Generalization Error vs. Number of Features Graph. **(D)** In the NAFLD group, LASSO regression analysis was used to screen out characteristic genes. **(E)** Generalization error versus number of features graph. **(F)** Intersection identifying NCAPH and IRS2 as final diagnostic markers.

In the validation sets (GSE151839 and GSE89632), Figure 3A demonstrated the expression levels of 134 shared DEGs between disease (obesity/NAFLD) and normal samples. The results indicated that there were 21 genes with significant differences (*P* < 0.05) between groups and consistent with the expression trend in the training sets (GSE59034 and GSE135251), thus, these 21 genes were considered as candidate genes. Immediately thereafter, based on the training sets (GSE59034 and GSE135251), these 21 candidate genes were fed into the LASSO and SVM-RFE methods. In GSE59034 dataset, seven feature genes (MR1, NCAPH, LAMB3, SQLE, CX3CR1, PFKFB3, and IRS2) were screened by LASSO with lambda.min = 0.0296 (Figure 3B) as well as the SVM-RFE selected seven feature genes (NCAPH, SQLE, SLC19A2, CX3CR1, IRS2, PFKFB3, and ACP5) with error rate of 0.113 and accuracy rate of 0.944 (Figure 3C). Meanwhile, in GSE135251 dataset, six feature genes (NCAPH, ITGB5, ACP5, SLCO2B1, MAP3K5, and IRS2) were screened from the LASSO algorithm (lambda.min = 1 × 10^–4^) and 21 feature genes were screened from the SVM-RFE algorithm (error rate = 0.00898 and accuracy rate = 0.991) in the same way, respectively (Figures 3D,E). Ultimately, the feature genes obtained by the above two methods were intersected, yielding two intersecting genes (NCAPH and IRS2), which were recorded as shared biomarkers in obesity and NAFLD (Figure 3F).

### 3.7 Shared biomarkers had excellent diagnostic values for obesity and NAFLD

**FIGURE 4:**
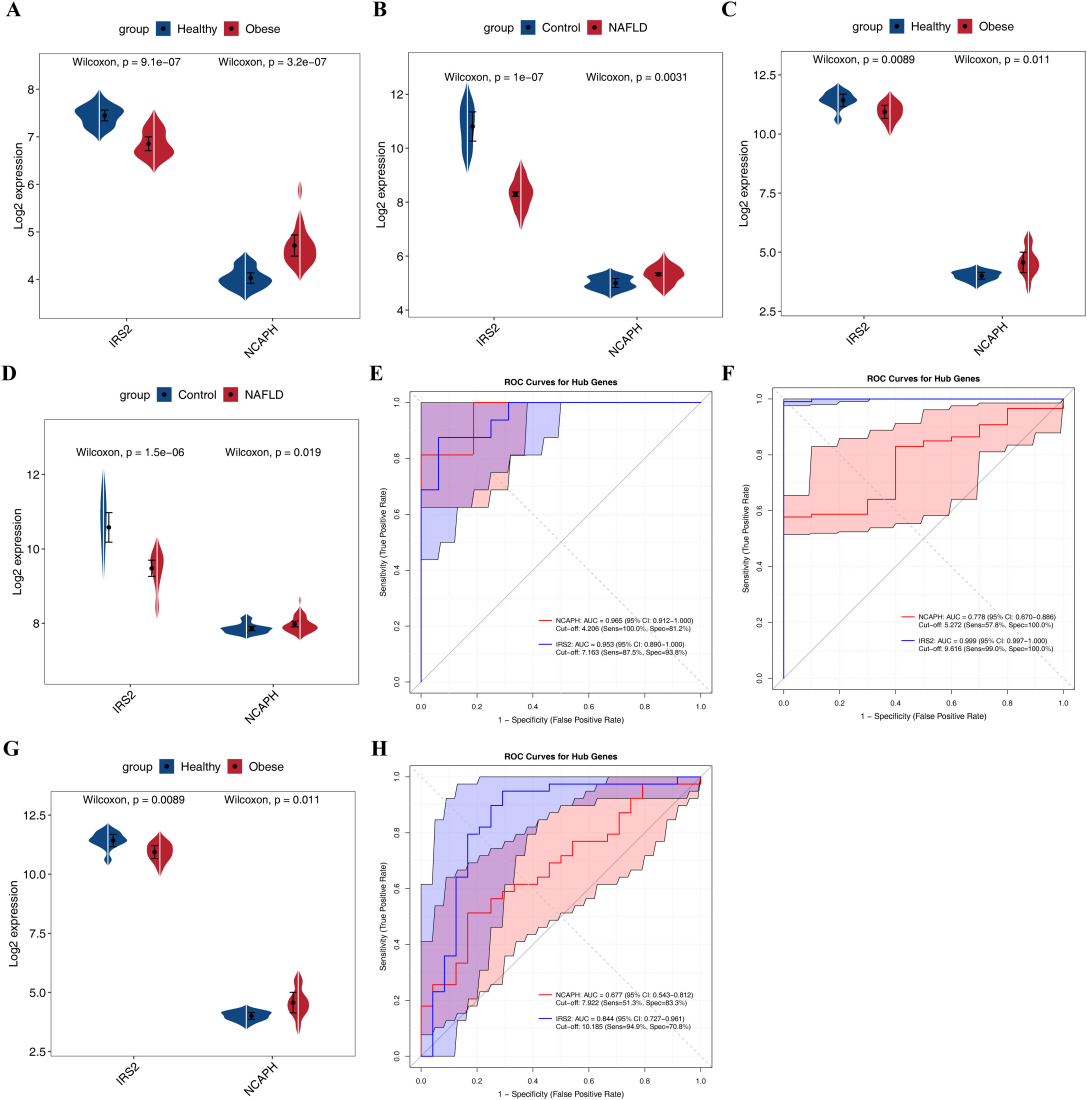
Validation and diagnostic efficacy of shared biomarkers. **(A–D)** Expression levels of NCAPH (up-regulated) and IRS2 (down-regulated) in training and validation sets. **(E–H)** ROC curves demonstrating high discriminative ability (AUC > 0.6) for both obesity and NAFLD.

In the training sets GSE59034 (obesity) and GSE135251 (NAFLD), NCAPH was significantly upregulated in the disease samples, while IRS2 was consistently downregulated, suggesting a stable and opposite regulatory pattern for both in obesity and NAFLD (Figures 4A,B). In the validation sets (GSE151839 and GSE89632), the expression trends were consistent with those in the training sets (Figures 4C,D). ROC curve analysis revealed that in the obesity-related datasets (GSE59034 and GSE151839), the AUC values of NCAPH were 0.965 and 0.83, and those of IRS2 reached 0.953 and 0.84; in the NAFLD-related datasets (GSE135251 and GSE89632), the AUC values of NCAPH were 0.778 and 0.677, and IRS2 was even as high as 0.999 and 0.844 (Figures 4E–H). All AUC values exceeded 0.6 and most were close to or higher than 0.8, indicating excellent discriminatory efficacy for disease and normal samples for both, especially the AUC value of IRS2 in the obesity training set was close to 1, suggesting its potential as a diagnostic indicator.

### 3.8 An effective nomogram model was created

**FIGURE 5:**
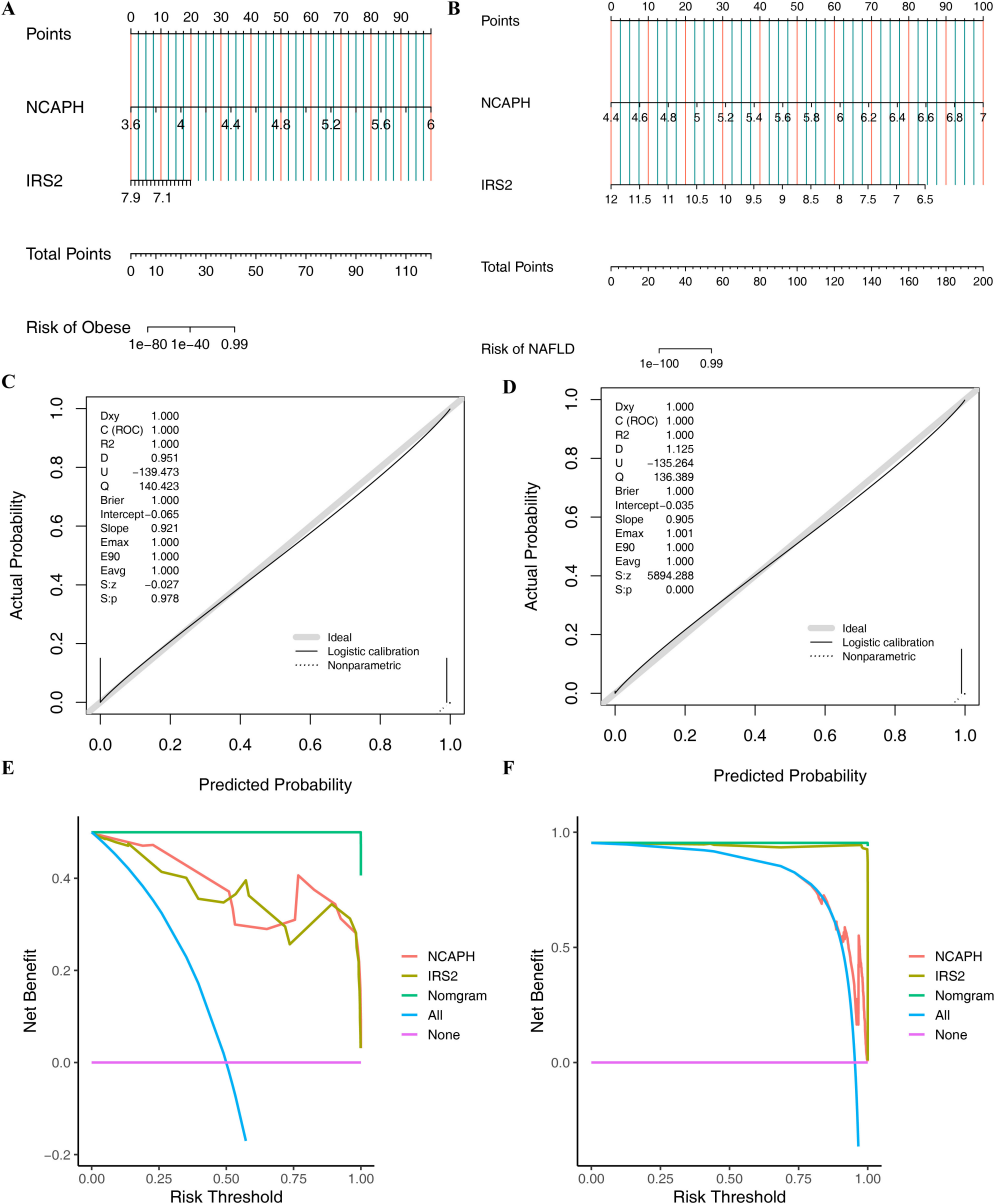
Diagnostic nomogram construction and evaluation. **(A,B)** Nomograms predicting obesity and NAFLD risk based on NCAPH and IRS2. **(C,D)** Calibration curves showing agreement between predicted and actual probabilities. **(E,F)** DCA curves indicating high clinical net benefit.

In training sets (GSE59034 and GSE135251), the nomograms based on two shared biomarkers were constructed correspondingly, providing a reliable tool for clinical diagnosis of obesity and NAFLD (Figures 5A,B). Besides, the calibration curves matched well with the ideal curves, implying that the ability of the nomograms diagram was reliable for predicting the occurrence of obesity and NAFLD (Figures 5C,D). Meanwhile, it could be observed from the decision curve that the clinical benefits of the nomograms were higher than that of the individual shared biomarkers, which meant that the constructed nomograms had high clinical reference values for predicting obesity and NAFLD (Figures 5E,F).

### 3.9 Enrichment analysis was completed of shared biomarkers in obesity and NAFLD

**FIGURE 6:**
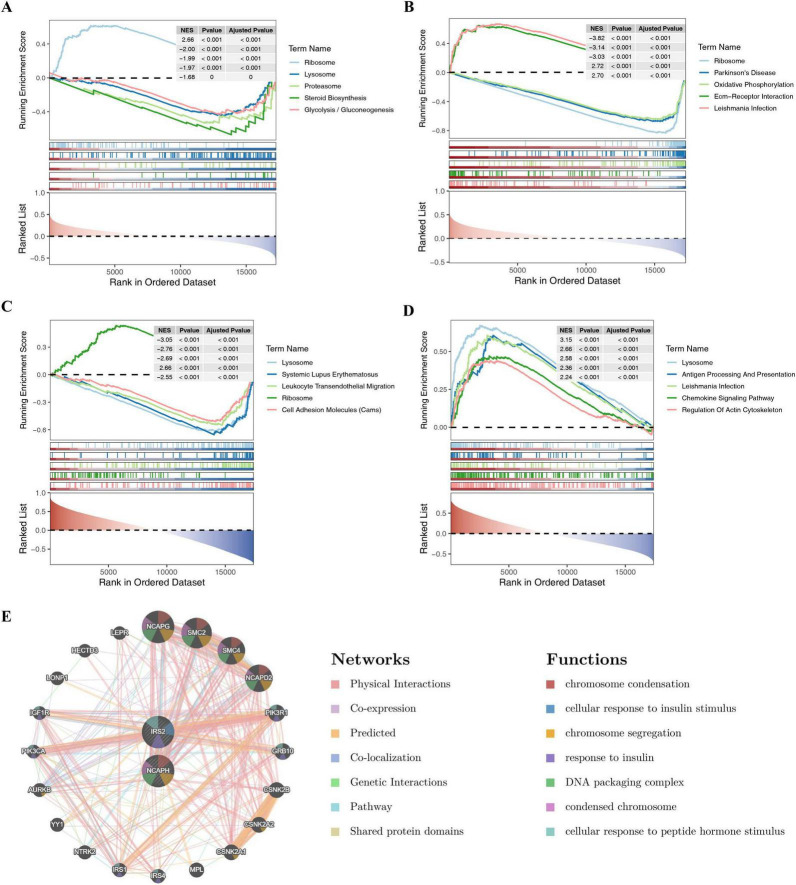
Single-gene GSEA and interaction network. **(A–D)** Significant KEGG pathways enriched in NAFLD **(A,B)** and obesity **(C,D)** samples. **(E)** GeneMANIA network showing co-expression and physical interactions (e.g., with NCAPG, SMC2).

To explore the underlying biological mechanisms of shared biomarkers, GSEA was accomplished in the training sets (GSE59034 and GSE135251). In patients with NAFLD, the KEGG gene set was found that NCAPH and IRS2 were all significantly enriched in these pathways, such as “ECM-receptor interaction,” “TGF-beta signaling pathway,” “p53 signaling pathway,” “MAPK signaling pathway,” “Jak-STAT signaling pathway,” “Cytokine-cytokine receptor interaction,” etc (Figures 6A,B). In patients with obesity, NCAPH and IRS2 were mostly co-enriched for “Chemokine signaling pathway,” “Fc gamma R-mediated phagocytosis,” “Cytokine-cytokine receptor interaction,” “NOD-like receptor signaling pathway,” “Toll-like receptor signaling pathway,” “ECM-receptor interaction,” “Jak-STAT signaling pathway” and other pathways (Figures 6C,D). These results emphasized that two shared biomarkers were involved in multiple biological pathways that might contribute to the progression of obesity and NAFLD.

Furthermore, the functional interaction network was generated through GeneMANIA database to further investigate shared biomarkers. Based on the findings, shared biomarkers seemed to interact significantly with NCAPG, SMC2, SMC4, and NCAPD2, as well as these genes were involved in biological functions such as “chromosome condensation,” “cellular response to insulin stimulus,” “response to insulin,” etc (Figure 6E).

### 3.10 Chromosomal localization, subcellular localization, and potential regulatory analyses were completed

**FIGURE 7:**
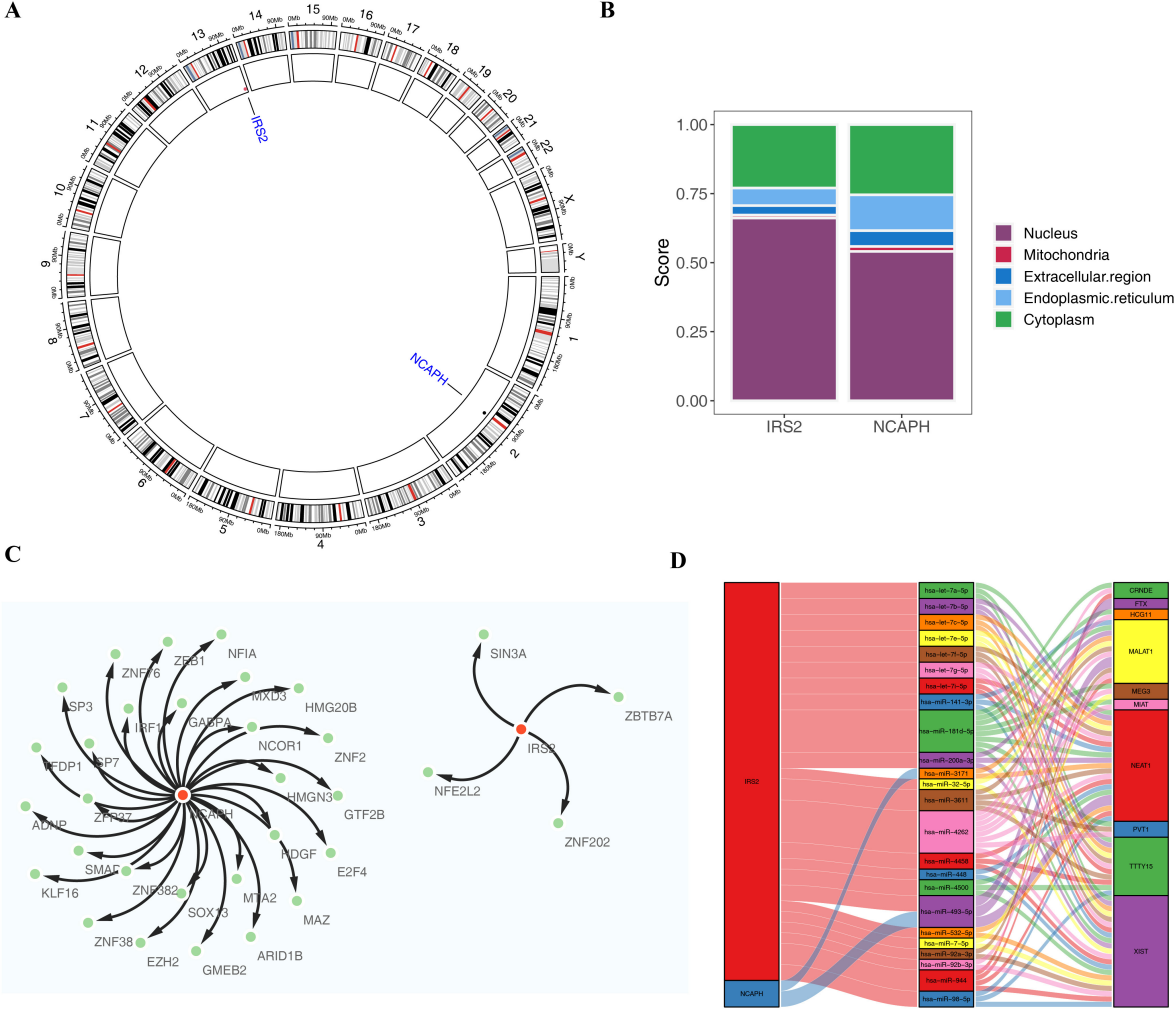
Chromosomal localization and regulatory networks. **(A,B)** Chromosomal distribution and subcellular localization of NCAPH and IRS2. **(C)** TF-mRNA regulatory network. **(D)** ceRNA network illustrating upstream regulation by lncRNAs (e.g., NEAT1, MALAT1).

The results of chromosomal localization analysis revealed that NCAPH and IRS2 were located in chromosomes 2 and 13, respectively (Figure 7A). Meanwhile, the three biomarkers were entered into the mRNALocater database to analyze their subcellular localization, which indicated that NCAPH and IRS2 both showed high expression in the nucleus and cytoplasm (Figure 7B).

Two shared biomarkers were entered into the ENCODE database, yielding a total of 32 TFs of regulatory shared biomarkers to elucidate the regulatory mechanisms of shared biomarkers. The TF-mRNA network contained 34 nodes and 32 edges, of which NCAPH was regulated by 28 TFs (MTA2, GABPA, etc.) and IRS2 was regulated by four TFs (ZBTB7A, NFE2L2, etc.) (Figure 7C). Furthermore, based on the Starbase, miRDB and miRcode databases, a lncRNA-miRNA-mRNA network was constructed, which consisted of two shared biomarkers (NCAPH and IRS2), 24 miRNAs and 10 lncRNAs (Figure 7D). Obviously, the multiple lncRNA-miRNA-mRNA relationship pairs were found in this network, such as, lncRNAs (NEAT1, MALAT1, and FTX) could simultaneously regulate two shared biomarker via miRNA (hsa-miR-493-5p), as well as hsa-let-7 family members (hsa-let-7a-5p, hsa-let-7b-5p, hsa-let-7c-5p, hsa-let-7e-5p, hsa-let-7f-5p, hsa-let-7g-5p, and hsa-let-7i-5p) were identified as regulators of IRS2.

### 3.11 Experimental verification of shared biomarkers in obesity and NAFLD

**FIGURE 8:**
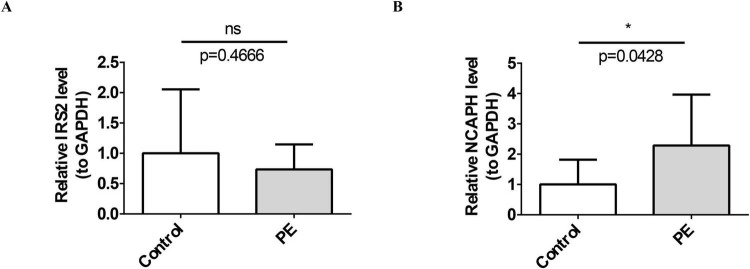
Experimental validation. qRT-PCR confirms significant up-regulation of NCAPH in clinical obesity and NAFLD samples, consistent with bioinformatics predictions. **(A)** The expression level of IRS2. **(B)** The expression level of NCAPH. ns indicates not significant; * indicates *P* < 0.05.

With the purpose of verifying demonstrate the expression of shared biomarkers in obesity and NAFLD samples, qRT-PCR was performed. As shown in Figure 8. the expression of NCAPH in diseased tissue was significantly upregulated, and the result was consistent with the bioinformatics analysis. However, although IRS2 showed an expression trend consistent with the bioinformatics analysis, there was no significant difference in expression levels between diseased and healthy tissues.

## 4 Discussion

The obesity epidemic is closely associated with the rising prevalence and severity of NAFLD (30). This study identified the causal relationship between obesity and NAFLD through MR. The differential genes shared by obesity and NAFLD were identified through transcriptomics, and the biological pathways and regulatory networks they are involved in were analyzed. Furthermore, RT-qPCR was used to verify the consistency between the expression of biomarkers in clinical samples and the bioinformatics results. The study aims to further understand the potential mechanisms by which obesity develops into NAFLD at the genetic level. These findings may inform the future design of molecular diagnostics and the development of personalized treatment for patients with NAFLD and obesity.

Liu et al. showed that genetically driven NAFLD significantly increases the risk of centripetal obesity (31). Evidence from a meta-analysis of 21 cohort studies suggests that obese individuals have a 3.5-fold increased risk of NAFLD, with a significant dose-dependent relationship between BMI and NAFLD risk (32). Our findings suggest that obesity increases the risk of developing NAFLD by 25 percent, which is consistent with the trend of previous findings. Possible explanations for this relationship are as follows. Abnormal lipid metabolism in obese patients is involved in the development of NAFLD (33). Specifically, an imbalance between lipid uptake, catabolism, production, oxidation, and secretion leads to excessive lipid accumulation in the liver, which further contributes to endoplasmic reticulum stress, mitochondrial dysfunction, lysosomal dysfunction, extracellular vesicle secretion, and aggravated hypoxia (34, 35). Dysregulated lipid metabolism creates a lipotoxic environment that promotes the development of NAFLD (36). There is a strong relationship between intestinal flora and obesity and NAFLD (37). Decreased abundance of bifidobacteria and decreased ratio of Proteobacteria to Firmicutes in patients with NAFLD and obesity are risk factors for the progression of NAFLD. Imbalance of bacterial flora causing metabolic endotoxemia, perturbation of bile acid metabolism, production of endogenous ethanol and other toxic products play an important role in the pathogenesis of NAFLD (38–40). In addition, the increased severity of NAFLD in obese patients is strongly associated with impaired hepatic mitochondrial fatty acid oxidation and mitochondrial turnover (41, 42).

Of note, the Mendelian randomization (MR) analysis in the present study was based on sex-unstratified genome-wide association study (GWAS) summary data, thereby precluding the evaluation of the potential modifying effect of sex on genetic effects, and accumulating evidence has demonstrated remarkable sexual dimorphism in the prevalence, clinical phenotypes, and pathological mechanisms of obesity and non-alcoholic fatty liver disease (NAFLD) (43); for instance, estrogen exerts metabolic protective effects in premenopausal females (44, 45), and several obesity-associated genes (e.g., FTO) and NAFLD-susceptible genes (e.g., PNPLA3) may exert differential effects across sexes (46–48), accordingly, the current analysis may have masked the sex heterogeneity of the causal relationship between obesity and NAFLD; furthermore, this study primarily focused on the intrinsic mechanisms at the genetic and transcriptomic levels, without incorporating the influence of critical environmental factors, particularly infectious agents, for example, Adenovirus type 36 (Ad-36) is a virus conclusively associated with obesity and metabolic dysregulation in humans to date (49, 50), and represents a well-characterized biological factor among the environmental etiologies of obesity (51, 52), which can promote obesity and hepatic steatosis via an adipocyte-dependent pathway and may regulate lipid metabolic pathways through epigenetic mechanisms (53, 54), thereby interacting with the genetic background to influence the onset and progression of NAFLD, and future studies are warranted to perform MR analyses using sex-stratified GWAS data to uncover the sex-specific genetic effects within the obesity–NAFLD causal cascade, while integrating environmental exposure data such as viral serology or molecular detection will facilitate in-depth investigations into the interaction between environmental factors (e.g., Ad-36) and genetic susceptibility at both epidemiological and mechanistic levels, which will contribute to a more comprehensive and systematic dissection of the multifactorial etiological network of NAFLD.

Insulin binds to the insulin receptor (IR) and activates its tyrosine kinase, which promotes tyrosine kinase phosphorylation of IR substrates (IRS), such as IRS-1 and IRS-2, both of which are abundantly expressed in hepatocytes (11). Honma et al. found that the expression of IRS2 was reduced in patients with NAFLD (55), which is in agreement with our findings. And preferential downregulation of IRS-2 may contribute to selective resistance to the inhibitory effect of insulin on gluconeogenesis. IRS2 regulates the PI3K/AKT/FOXO1 pathway, which controls the repression of gluconeogenic genes, and IRS2 promotes insulin signaling (56). Hippo signaling, by regulating IRS2 expression in interaction with AKT signaling interaction can prevent NAFLD (57). In addition, LncARSR was found to promote the progression of NAFLD by reducing YAP1 phosphorylation activating the IRS2/AKT pathway, which further increased lipid accumulation, cell proliferation, invasion and cell cycle (58). In contrast, NCAPH is highly expressed in both obese and NAFLD patients. However, unfortunately, NCAPH gene has not yet been explored in obesity and NAFLD, we will do further research and validation afterward, and advocate more studies focusing on this gene and the potential mechanism of action behind it.

Through GSEA in this study, it was found that NCAPH and IRS2, the shared biomarkers of obesity and NAFLD, were both significantly enriched in the “Cytokine-cytokine receptor interaction,” “ECM-receptor interaction,” and “Jak-STAT signaling pathway.” Both genes can regulate these three core pathways to jointly contribute to the pathological progression of the two diseases. Specifically, in the “Cytokine-cytokine receptor interaction” pathway, NCAPH can mediate liver immune cell infiltration associated with inflammatory factors, thereby promoting obesity-related chronic low-grade inflammation in the liver. In the “ECM-receptor interaction” pathway, NCAPH modulates the interaction between hepatocytes and the extracellular matrix, influencing the fibrotic remodeling of liver tissue—this is consistent with the pathological features of hepatic stellate cell activation and collagen deposition in NAFLD. In the “Jak-STAT signaling pathway,” NCAPH transmits inflammatory signals to amplify local inflammatory responses in the liver, which aligns with the role of this pathway as a crosstalk node between inflammation and metabolic disorders in metabolic liver diseases.

For IRS2, in the “Cytokine-cytokine receptor interaction” pathway, it balances the release of inflammatory factors and metabolic homeostasis, preventing excessive inflammation from disrupting hepatic metabolic function. In the “ECM-receptor interaction” pathway, IRS2 regulates the expression of extracellular matrix components in liver tissue to inhibit excessive fibrosis. In the “Jak-STAT signaling pathway,” IRS2 participates in the crosstalk regulation of insulin and inflammatory signals, maintaining the balance of hepatic glucose and lipid metabolism—this is consistent with the regulatory role of this pathway in insulin resistance. Collectively, by collectively regulating these three core pathways linking “inflammation-fibrosis-metabolism,” NCAPH and IRS2 exert a synergistic effect in obesity and NAFLD. They not only participate in the activation and balance of inflammation as well as the fibrotic remodeling of liver tissue but also maintain metabolic homeostasis. These findings provide key insights for deciphering the common molecular mechanisms of the two diseases and lay a theoretical foundation for the subsequent development of therapeutic strategies targeting these genes and related pathways.

This study revealed that the upregulation of NCAPH combined with the downregulation of IRS2 serve as shared biomarkers for obesity and NAFLD, whose diagnostic performance (with AUC consistently > 0.67) is not only statistically significant but also associated with distinct pathological mechanisms. As a key adaptor protein in insulin signaling, the downregulation of IRS2 is directly linked to hepatic insulin resistance and disorders of glucose and lipid metabolism (59, 60); consistently low expression of IRS2 was observed across multiple cohorts in the present study, further supporting the loss of its protective role in the pathogenesis of NAFLD. In contrast, NCAPH is a chromosome condensation protein (61), and its upregulation may reflect abnormal cell cycle progression or enhanced DNA damage stress in the context of obesity and NAFLD. Notably, in addition to intrinsic metabolic and cell cycle disturbances, environmental factors such as viral infection have also been proposed to contribute to the development of obesity and NAFLD. The favorable discriminatory power demonstrated by these two molecules in ROC analysis provides a potential basis for their translation into non-invasive diagnostic indicators. Collectively, NCAPH and IRS2 not only possess diagnostic value as biomarkers but their expression alterations also point to multi-layered mechanisms including impaired insulin signaling, dysregulated cell cycle progression, and interactions with potential environmental factors, offering novel insights into the molecular link underlying the progression from obesity to NAFLD.

Verification results via qRT-PCR showed that the expression of NCAPH was completely consistent with the bioinformatics analysis results: it was significantly upregulated in obesity and NAFLD samples. Moreover, ROC curve analysis confirmed its favorable diagnostic efficacy, which further verified the reliability of NCAPH as a shared biomarker for obesity and NAFLD, supporting its use as a core target for subsequent in-depth studies. Regarding IRS2, although qRT-PCR results exhibited a downregulated expression trend consistent with the bioinformatics findings, no statistically significant difference was detected between disease samples and healthy control samples. Existing studies have confirmed that myeloid IRS2 deletion can enhance sympathetic nerve function in adipose tissue and restrict obesity progression; furthermore, in NAFLD, activation of the IRS2/AKT pathway effectively inhibits hepatic lipid accumulation in NAFLD model mice, highlighting the important therapeutic value of IRS2 in disease treatment. The qRT-PCR results of the present study showed a downward trend in the expression of IRS2 in the disease group, although the difference did not reach statistical significance. Beyond sample-size considerations, this observation is more likely to reflect the complexity of biological regulation, as IRS2 expression is jointly influenced by tissue and cellular heterogeneity, disease-stage dependence, and feedback modulation of the insulin signaling pathway, leading to considerable variability in overall mRNA levels (62–64). Despite the non-significant changes in tissue mRNA levels, multiple lines of evidence support the critical role of IRS2 in the pathogenesis of both diseases. The integrated analysis of the present study and previous literature indicates that IRS2 serves as a key regulator of insulin signaling and metabolic homeostasis, and its dysfunction is directly linked to insulin resistance (65); moreover, the downward trend observed herein is consistent with bioinformatic predictions. As a core mediator of insulin signaling, IRS2 expression is likely closely associated with individual metabolic phenotypes. We hypothesize that significant downregulation of IRS2 may occur specifically in subtypes characterized by more severe insulin resistance. Future studies incorporating clinical parameters such as blood glucose and insulin levels are warranted to further clarify the diagnostic and subtyping value of IRS2, thereby facilitating precision diagnosis and targeted therapy tailored to distinct pathophysiological features.

This study has several limitations. First, it was mainly based on public genomic and transcriptomic data from European populations, so the generalizability of the conclusions needs to be validated in multi-ethnic cohorts; the small sample size used for clinical validation may compromise statistical power. Second, although Mendelian randomization analyses suggested a causal effect of obesity on NAFLD, the potential pleiotropy of instrumental variables cannot be completely ruled out. In addition, the biomarkers (NCAPH and IRS2) identified by bioinformatics analysis and their predicted regulatory networks have not been sufficiently verified by functional experiments, and their specific molecular mechanisms remain unclear. Furthermore, IRS2 showed no significant difference in qRT-PCR validation, requiring further confirmation of its reliability as a biomarker. Finally, due to the limitations of current public databases, this study did not include the association analysis of environmental exposure factors such as Adenovirus-36 infection with NAFLD, which may somewhat limit the comprehensive understanding of the disease pathogenesis.

Future studies should validate the diagnostic value of NCAPH and IRS2 in multi-center, multi-ethnic large-scale clinical cohorts; clarify the specific mechanisms of these two genes in lipid metabolism and liver inflammation/fibrosis through functional experiments using cellular and animal models; systematically dissect their regulatory networks by integrating multi-omics data; and explore their clinical translational potential as non-invasive diagnostic biomarkers or therapeutic targets. Moreover, a more comprehensive set of environmental and infectious factors, including Adenovirus-36, should be incorporated to more fully delineate the pathogenic network underlying obesity and NAFLD. Through interdisciplinary collaboration and data sharing, these findings are expected to be translated into precise risk prediction and targeted intervention strategies.

This study is the first to successfully identify two key biomarkers—NCAPH and IRS2—in OBESITY and NAFLD using bioinformatics techniques and Mendelian randomization analysis. The results indicate that these two genes play significant roles in the onset and progression of OBESITY and NAFLD, and may potentially become targets for future disease diagnosis and treatment. Moreover, the study has constructed a related regulatory network, offering a new perspective for understanding the molecular mechanisms of obesity and NAFLD. Although further experimental mechanism research and clinical application studies are needed to validate these findings, we will continue to monitor the functions of these genes and explore their potential applications in disease diagnosis and treatment.

## Data Availability

The datasets presented in this study can be found in online repositories. The names of the repository/repositories and accession number(s) can be found below. The transcriptomic datasets analyzed during the current study are available in the Gene Expression Omnibus (GEO) repository under accession numbers GSE59034, GSE135251, GSE151839, and GSE89632. The summary statistics for obesity and NAFLD are available in the IEU Open GWAS project (https://gwas.mrcieu.ac.uk/) under the IDs finn-b-E4_OBESITY and finn-b-NAFLD. The clinical raw data supporting the qRT-PCR validation results are available from the corresponding author on reasonable request, as they contain information that could compromise the privacy of research participants.

## Footnotes

1
http://www.ncbi.nlm.nih.gov/geo/

2
http://www.ehbio.com/test/venn/#/

3
https://STRING-db.org/

4
http://www.broadinstitute.org/gsea/msigdb/index.jsp

5
https://genemania.org

6
http://circos.ca/

7
http://bio-bigdata.cn/mRNALocater

8
https://maayanlab.cloud/Harmonizome/dataset/ENCODE+Transcription+Factor+Targets

9
http://starbase.sysu.edu.cn/

10
https://mirdb.org/

11
http://www.mircode.org/

## Supplementary material

Supplementary Table 1 The table of clinical sample information for the experiment.

Supplementary Table 2 Primer sequences used for qt-PCR.

Supplementary Table 3 Reaction system components for qRT-PCR.

Supplementary Table 4 Thermal cycling conditions for qRT-PCR.
